# Understanding the differences in occupational injuries due to accidents among native-born and immigrant workers in Sweden: a repeated cross-sectional register-based study

**DOI:** 10.1186/s40621-025-00616-7

**Published:** 2025-09-08

**Authors:** Devy L. Elling, Theo Bodin, Helena Honkaniemi, Bertina Kreshpaj, Letitia Davis, Alicia Nevriana, David H. Wegman, Eskil Wadensjö, Katarina Kjellberg, Nina Bilal, Emelie Thern

**Affiliations:** 1 Unit of Occupational Medicine, Institute of Environmental Medicine, Karolinska Institutet, Stockholm, SE-171 77 Sweden; 2 Centre of Occupational and Environmental Medicine, Region Stockholm, Stockholm, SE-113 65 Sweden; 3 Department of Public Health Sciences, Stockholm University, Stockholm, SE-106 91 Sweden; 4 Copenhagen Health Complexity Center, Department of Public Health, University of Copenhagen, Copenhagen, DK-1353 Denmark; 5 Massachusetts Department of Public Health, Boston, MA, Massachusetts 02108 USA; 6 Unit of Integrative Epidemiology, Institute of Environmental Medicine, Karolinska Institutet, Stockholm, SE-171 77 Sweden; 7 Department of Public Health, University of Massachusetts Lowell, Lowell, MA, Massachusetts 01854 USA; 8 Swedish Institute for Social Research, Stockholm University, Stockholm, SE-106 91 Sweden; 9 Department of Social Sciences, Technology and Arts, Division of Humans and Technology, Luleå University of Technology, Luleå, SE-971 87 Sweden

**Keywords:** Occupational accident, Repeated cross-sectional study, Immigrant, Register-based study

## Abstract

**Background:**

Immigrants continue to face challenges after entering the labor market and remain overrepresented in ‘3-D jobs’ (dirty, difficult, degrading). This study aims to investigate the differences in occupational injury due to accidents (OIA) among immigrants compared to native-born workers in Sweden, and to examine the role of migrant-specific and work factors in these differences.

**Methods:**

This repeated cross-sectional study used nationwide registers including all gainfully employed individuals in 2004–2020 (average annual sample 4.5 million individuals). OIA was treated as a binary outcome and migrant status was categorized based on region of birth and reason for immigration. OIA odds were estimated using pooled logistic regression analyses, where the crude model was adjusted for sociodemographic factors, time since immigration, and work factors.

**Results:**

First-generation immigrants (odds ratios [OR] 1.41; 95% confidence interval [CI] 1.40, 1.42) and second-generation immigrants (OR 1.10; 95% CI 1.09, 1.11) had higher odds of OIA than native-born workers. Among the first-generation immigrants, the strength of the association varied depending on region of birth and reason for immigration. Immigrating to Sweden for work reasons was associated with lower odds of OIA among first-generation immigrants (OR 0.62; 95% CI 0.61, 0.64). The elevated odds of OIA among immigrants relative to native-born workers remained after adjusting for important covariates.

**Conclusions:**

The differences in OIA underscore the disparities among native-born and immigrant workers in Sweden. The current findings highlight the importance of addressing these issues to ensure a safe work environment for all.

**Supplementary Information:**

The online version contains supplementary material available at 10.1186/s40621-025-00616-7.

## Background

Globally, an estimated 245 million people (older than 15 years) were not living in their home country in 2019, with 169 million classified as migrant workers [1]. Many high-income countries are expected to have an increase in the immigrant population in the future. As the migrant population settles in the host country (henceforth ‘immigrants’), partaking in the labor market is considered crucial for their integration [2].

In Sweden, approximately 20% of the population was estimated to have a foreign background in 2023, of which most are of working age (25–34 years) [3]. The participation of immigrants in the Swedish labor market is becoming increasingly important, as they contribute to economic outputs and may address labor shortages due to aging of the native-born population [1, 4]. Despite a growing demographic, immigrants are met with challenges, such as language barriers, a mismatch between skills and job demand in the host country [5] and lack of social network with the native-born population [1, 6]. These challenges often vary by the reason for immigration and time since immigration [4, 7].

Immigrants are disproportionately represented in so-called ‘3-D jobs’ (dirty, dangerous, and difficult) [8–10]. These jobs often involve labor-intensive work and are concentrated in informal and/or less regulated sectors, such as agriculture [1, 11]. As such, immigrants are more likely to experience a poorer work environment compared to native-born workers, characterized by higher physical demands, lower job control and lower support [8, 10]. Consequently, immigrant workers are more likely to have an increased risk of occupational injuries due to accidents (OIA).

An earlier Swedish report estimated a 44% higher risk of OIA among immigrant workers compared to native-born; however, this report only examined immigrants as one homogeneous group [12]. A more recent Swedish study examining OIA trends by comparing first-generation immigrant groups found an overall higher incidence rate of OIA among first-generation immigrant workers, with some variations based on region of origin and reason for immigration compared to native Swedes, despite a relatively stable or a slightly declining trend between 2003 and 2020 [13]. This was consistent with literature in international settings [8, 10, 14]. For example, a Danish study found a 57% increased risk of OIA among immigrants from outside of the European Union (EU) [14]. Region of birth, often correlated with reason for immigration, may also explain some of these differences [7]. In addition, time since immigration may influence the type of work accessible for immigrants [7]. Newly arrived immigrants may have limited opportunities to get jobs that match their skills due to waiting time to receive work permits or high language requirements in the host country [4, 5, 15].

Despite consistent evidence on the elevated risk of OIA among immigrants [10, 14, 16], little is known about factors that could explain these differences. Most studies continue to focus mainly on individual-level determinants, such as country of origin and time since immigration [7, 10]. Work-related risk factors that may contribute to these risks remain largely unexplored. Common risk factors for OIA include frequent job changes and full-time employment [17]. Frequent job changes may reflect inconsistent work experience and limited awareness of workplace hazards, while full-time employment may lead to greater cumulative exposure to such occupational hazards due to longer working hours and sustained exposure over time [17]. Immigrants tend to change jobs more frequently and more likely to have part-time employment compared to native-born workers [4, 18]. It is plausible that immigrants are more likely to undertake undesirable or hazardous tasks at work compared to native-born workers, thereby increasing their exposure to workplace risks. An Italian study found that immigrants who work in construction had a two-fold higher occurrence of OIA compared to native-born workers when conducting the same type of work [19]. This suggests that immigrants may face more hazardous conditions even within the same industrial sector [4, 16].

This study builds on the recent findings on trends of OIA among immigrant workers in Sweden summarized above [13] by attempting to identify some of the understudied underlying risk factors for these disparities. The current study aims to investigate the differences in OIA among immigrants compared to native-born workers in Sweden, and to examine the role of migrant-specific and work factors in these differences.

## Methods

### Study design and study population

In the current study, individuals were included if they: (i) resided in Sweden, (ii) were gainfully employed with an income of at least 100 Swedish kronor at the end of each year, (iii) had full information regarding migration status (for the immigrant population), and (iv) did not emigrate, immigrate, or die during the year of interest. We set the lowest level of income to be able to include as many individuals as possible. Swedish-born who emigrated and returned to Sweden were excluded as these individuals represent a uniquely different sub-population of immigrants. Individuals were included in each year (2004 to 2020) in which the inclusion criteria were met, with the possibility to contribute to multiple years. OIA and migration status were measured every year, resulting in an average sample of approximately 4.5 million individuals annually.

### Data source

This register-based repeated cross-sectional study was based on Swedish data from 2004 to 2020. Information about the study population was gathered from three data sources: (i) the Longitudinal Integrated Database for Health Insurance and Labor Market Studies (LISA), which contains information on workers’ sociodemographic characteristics, occupation, industrial sector, and employment status; (ii) the Longitudinal Database for Integration Studies (STATIV), which comprises data on migration status, including reason for immigration and year of immigration; and (iii) the Information System on Occupational Injuries (ISA), provided by the Swedish Work Environment Authority containing information on OIA and severity estimated by the number of days on sick leave. These nationwide registers were linked through the unique personal identity number in Sweden. The personal identity numbers are granted to individuals who intend to stay in Sweden for at least one year [15, 20]. Subsequently, undocumented immigrants and those with irregular administrative status are not included in the current study. Thereafter, a new unique and untraceable identification number was replaced by Statistics Sweden to ensure the confidentiality of the information.

### Variables

#### Occupational injuries due to accidents

OIA was identified from ISA, which was defined based on Swedish law as follows, “*an injury due to accident[s]*,* which occurred at the workplace or other place where the injured person had been for work. For an event to be counted as an accident*,* it is required that the course was relatively short and arose in connection with a particular event*”, which includes the way to-and-from work [21]. In Sweden, employees are responsible to report any OIA occurrences, and subsequently, the employer is legally responsible to report it to the Swedish Work Environment Agency [22, 23]. However, in cases where employers fail to do so, the employees themselves are able to report any incident in consultation with the work environment and safety officer [22]. In this study, the occurrence of OIA was dichotomized (being injured vs. not being injured) for each year included in the study. In cases where individuals reported multiple OIA in a single year, the most severe injury based on the number of reported sick leave days was retained. Severe injuries are less likely to be under-reported [24]. Fatal OIA was excluded from this study due to a small number of cases in our dataset.

#### Migration status

Migration status was defined based on the available information from the STATIV register. First, the overall migration status was categorized into three categories: native-born (born in Sweden with two Swedish-born parents), second-generation immigrants (born in Sweden with at least one foreign-born parent), and first-generation immigrants (born outside of Sweden with two foreign-born parents). Individuals with two native-born Swedish parents but who were born outside of Sweden comprise a small and diverse group and were excluded in the analyses.

To account for between-group variation among the first-generation immigrants [25], first-generation immigrants were further categorized based on region of birth (seven regions: Nordic or European Union [EU]/European Free Trade Association [EFTA] Member States, non-EU/EFTA European countries, North America/Oceania, Africa, Asia, Middle East, Other). EFTA countries included Iceland, Liechtenstein, Norway and Switzerland [26], whereas Other consisted of countries in Central and South America/Caribbean and unknown. Finally, first-generation immigrants were also categorized based on the reason for immigration (three reasons: work, family reunification or humanitarian, other). Native-born served as the reference group in all analyses. First-generation immigrants from the Nordic countries or EU/EFTA Member States were expected to have missing reason for immigration due to the free border movements between countries.

#### Covariates

The covariates included sociodemographic characteristics (sex, age, highest level of education), gathered from LISA for each year. Due to administrative delays, the highest educational levels (three categories: primary, secondary, tertiary) among first-generation immigrants could include information from the following year if individuals met the inclusion criteria. Time since immigration was calculated by subtracting the year of immigration from the year of interest (2004–2020), which was then categorized into four categories (1–5 years, 6–10 years, 11–20 years, 20 + years). This reflected elapsed time since the most recent immigration event, in which second-generation immigrants may have non-zero values if they emigrated and subsequently returned to Sweden. Additional covariates included job change in the past year, industry risk level (two categories: high-risk industry, low-risk industry), and full-time employment (yes/no). Information on job changes in the past year was operationalized through the change in the company’s registration number, which was also gathered from LISA. Information on high-risk industry was collected from reports published by the Swedish Work Environment Authority, which was categorized based on industry specific OIA rates [27–30]. The industry with an OIA rate of ≥ 25% higher than the national average was considered a high-risk industry. A list of high-risk industries in the study can be found in the supplementary materials (Table S1). Full-time employment to account for time-at-risk was not readily available in the dataset. The estimated extent of employment using a proxy variable, which was calculated using the median income based on sex, age strata, education level, residential region, and industry affiliation in Sweden for each year. Individuals were considered to have full-time employment if their income was at least 75% of the estimated median income in their strata. To ensure model robustness, multicollinearity using variance inflation factor (VIF) was assessed including all covariates, with all values below 3, indicating an acceptable level of collinearity.

### Statistical analysis

The distribution of characteristics of included individuals were presented as frequencies and percentages.

The crude and adjusted odds ratio (OR) with 95% confidence intervals (CI) were obtained using pooled multivariable logistic regression models for the study period (2004–2020). Cluster-robust standard errors, clustered at the individual level, were used to account for intra-individual correlation arising from multiple measurements of the same individual. In the analyses, separate analyses for each of the categories to define migrant status (overall migrant status, first-generation immigrants based on region of birth, first-generation immigrants based on reason for immigration) were performed. In the crude model no adjustments were done. Model 1 was adjusted for sex, age and education. Model 2 included covariates from Model 1 and was additionally adjusted for time since immigration. Model 3 included covariates from Model 2 with additional adjustments for job change, high-risk industry and full-time employment. In the analysis looking at reason for immigration, the model was not adjusted for time since immigration because of the large proportion of missing among first-generation immigrants from the Nordic or EU/EFTA Member States. In all the analyses, missing observations were included as a separate category in the analyses, ranging from 0.2% in education among native-born to 92.3% in time since immigration among second-generation immigrants.

Several sensitivity analyses were conducted. First, only individuals with the most severe OIA that required at least 14 days of sickness absence as the main outcome were included. This was done to reduce the bias of underreporting of OIA, which was compared to no occurrence of OIA. Second, second-generation immigrants were recategorized into workers with one native-born parent and workers with two immigrant parents. Third, the fully adjusted model in the main analyses was further adjusted for previous OIA during the previous year to examine whether the likelihood of OIA is higher among those who have reported a previous OIA. Finally, a complete case analysis was conducted, where individuals are included only if they have complete information on the covariates.

All analyses were performed using Stata version 17.

## Results

###  Sociodemographic characteristics by migration status

This study includes 7,110,408 unique individuals during the whole study period. Table 1 shows the characteristics of migration status for all individuals during the first year they fulfill the inclusion criteria. The majority of the study population were native-born (69.8%), and the smallest proportion were first-generation immigrants from North America or Oceania (0.4%). Compared to native-born workers, most immigrants are under 30 years of age and worked in service, care and sales. First-immigrants from North America or Oceania had the highest prevalence of individuals with tertiary level education (65.2%).

**Table 1:** Characteristics of included individuals by region of migration at first appearance in the cohort between 2004 and 2020 (*N* = 7,110,408)

	Native-born		Second-generation immigrants		First-generation immigrants													
					Nordic or EU/EFTA Member States		non-EU/EFTA European countries		North America or Oceania		Africa		Asia		Middle East		Other	
	*n*	%	*n*	%	*n*	%	*n*	%	*n*	%	*n*	%	*n*	%	*n*	%	*n*	%
Total	4,963,761	69.8	738,224	10.4	437,437	6.2	231,225	3.3	25,696	0.4	145,524	2.0	211,198	3.0	276,086	3.9	81,257	1.1
Sex																		
Male	2,544,193	51.3	374,421	50.7	217,576	49.7	125,553	54.3	14,783	57.5	84,062	57.8	102,390	48.5	168,386	61.0	40,846	50.3
Female	2,419,568	48.7	363,803	49.3	219,861	50.3	105,672	45.7	10,913	42.5	61,462	42.2	108,808	51.5	107,700	39.0	40,411	49.7
Age group																		
< 30 years	2,084,771	42.0	433,251	58.7	126,535	28.9	94,497	40.9	9955	38.7	67,731	46.5	106,161	50.3	121,929	44.2	32,612	40.1
31-40 years	902,214	18.2	138,509	18.8	111,591	25.5	66,859	28.9	8243	32.1	46,396	31.9	64,140	30.4	79,105	28.7	22,841	28.1
41-50 years	813,529	16.4	104,496	14.2	92,102	21.1	47,037	20.3	4795	18.7	23,286	16.0	30,196	14.3	53,272	19.3	15,891	19.6
51-60 years	854,347	17.2	52,902	7.2	80,434	18.4	18,986	8.2	2084	8.1	7018	4.8	9332	4.4	19,026	6.9	8238	10.1
>60 years	308,900	6.2	9066	1.2	26,775	6.1	3846	1.7	619	2.4	1093	0.8	1369	0.6	2754	1.0	1675	2.1
Education level																		
Primary	930,655	18.7	147,944	20.0	57,545	13.2	48,425	20.9	1341	5.2	46,928	32.2	49,955	23.7	75,466	27.3	12,705	15.6
Secondary	2,558,547	51.5	387,406	52.5	156,964	35.9	95,791	41.4	4809	18.7	54,800	37.7	59,094	28.0	95,642	34.6	32,902	40.5
Tertiary	1,466,725	29.5	199,829	27.1	177,706	40.6	73,892	32.0	16,764	65.2	37,071	25.5	82,385	39.0	96,783	35.1	33,001	40.6
Missing	7834	0.2	3045	0.4	45,222	10.3	13,117	5.7	2782	10.8	6725	4.6	19,764	9.4	8195	3.0	2649	3.3
Time since immigration																		
1-5 years	-	-	20,551	2.8	197,438	45.1	94,360	40.8	15,911	61.9	83,375	57.3	123,586	58.5	135,300	49.0	29,357	36.1
6-10 years	-	-	9262	1.3	31,860	7.3	46,866	20.3	2328	9.1	28,443	19.5	29,930	14.2	48,554	17.6	9279	11.4
11-20 years	-	-	14,454	2.0	57,827	13.2	60,869	26.3	3776	14.7	25,885	17.8	33,407	15.8	75,459	27.3	24,845	30.6
20+ years	-	-	12,787	1.7	150,243	34.3	29,075	12.6	3679	14.3	7762	5.3	24,214	11.5	16,617	6.0	17,762	21.9
Missing	-	-	681,170	92.3	69	0.0	55	0.0	2	0.0	59	0.0	61	0.0	156	0.1	14	0.0
Initial reason for immigration																		
Work	-	-	-	-	46,660	10.7	21,971	9.5	5471	21.3	5042	3.5	36,081	17.1	9539	3.5	5547	6.8
Family or humanitarian	-	-	-	-	57,283	13.1	149,257	64.6	10,958	42.6	118,014	81.1	113,806	53.9	235,213	85.2	44,068	54.2
Other	-	-	-	-	16,929	3.9	5680	2.5	1051	4.1	3943	2.7	13,225	6.3	3117	1.1	1837	2.3
Missing	-	-	-	-	316,565	72.4	54,317	23.5	8216	32.0	18,525	12.7	48,086	22.8	28,217	10.2	29,805	36.7
Job type																		
Military	15,489	0.3	1814	0.2	215	0.0	62	0.0	12	0.0	12	0.0	40	0.0	30	0.0	30	0.0
Managerial work	205,406	4.1	19,976	2.7	13,107	3.0	3240	1.4	1028	4.0	575	0.4	2287	1.1	1700	0.6	862	1.1
Work with requirement for in-depth university competence	673,049	13.6	81,609	11.1	75,287	17.2	21,855	9.5	8543	33.2	7339	5.0	27,112	12.8	21,442	7.8	10,280	12.7
Work with requirement for university education	705,185	14.2	84,526	11.4	47,567	10.9	13,175	5.7	2916	11.3	4532	3.1	11,633	5.5	13,277	4.8	6414	7.9
Administration and customer service	410,588	8.3	62,589	8.5	27,386	6.3	12,079	5.2	1321	5.1	6437	4.4	8817	4.2	13,423	4.9	4756	5.9
Service, care and sales	971,921	19.6	157,190	21.3	65,201	14.9	43,551	18.8	2844	11.1	44,540	30.6	46,851	22.2	68,707	24.9	19,682	24.2
Agriculture, gardening, forestry and fishing	51,311	1.0	3776	0.5	3352	0.8	1224	0.5	135	0.5	839	0.6	946	0.4	905	0.3	236	0.3
Constructions and manufacturing	381,966	7.7	43,137	5.8	35,493	8.1	15,975	6.9	939	3.7	3713	2.6	5090	2.4	11,327	4.1	3209	3.9
Machine manufacturing and transport	389,546	7.8	50,660	6.9	32,328	7.4	25,029	10.8	601	2.3	7367	5.1	9296	4.4	17,155	6.2	4639	5.7
Work with requirement for shorter training or introduction	296,892	6.0	52,749	7.1	39,480	9.0	35,762	15.5	1136	4.4	27,913	19.2	35,162	16.6	31,516	11.4	13,411	16.5
Missing	862,408	17.4	180,198	24.4	98,021	22.4	59,273	25.6	6221	24.2	42,257	29.0	63,964	30.3	96,604	35.0	17,738	21.8
High-risk industry																		
No	3,286,475	66.2	507,236	68.7	284,851	65.1	148,065	64.0	20,793	80.9	85,310	58.6	151,194	71.6	184,714	66.9	55,709	68.6
Yes	1,677,286	33.8	230,988	31.3	152,586	34.9	83,160	36.0	4903	19.1	60,214	41.4	60,004	28.4	91,372	33.1	25,548	31.4
Full-time employment																		
No	2,367,216	47.7	427,062	57.8	185,795	42.5	132,633	57.4	11,891	46.3	103,052	70.8	127,735	60.5	199,279	72.2	49,154	60.5
Yes	2,477,164	49.9	284,750	38.6	195,193	44.6	79,872	34.5	10,190	39.7	32,707	22.5	55,789	26.4	63,779	23.1	27,966	34.4
Missing	119,381	2.4	26,412	3.6	56,449	12.9	18,720	8.1	3615	14.1	9765	6.7	27,674	13.1	13,028	4.7	4137	5.1
Job change in the past year																		
No	3,067,634	61.8	360,367	48.8	191,102	43.7	90,012	38.9	7511	29.2	42,532	29.2	59,567	28.2	81,672	29.6	33,358	41.1
Yes	1,896,127	38.2	377,857	51.2	246,335	56.3	141,213	61.1	18,185	70.8	102,992	70.8	151,631	71.8	194,414	70.4	47,899	58.9
Occurrence of OIA																		
No	4,886,736	98.4	726,694	98.4	430,782	98.5	226,662	98.0	25,439	99.0	143,030	98.3	208,712	98.8	270,334	97.9	79,759	98.2
Yes	77,025	1.6	11,530	1.6	6655	1.5	4563	2.0	257	1.0	2494	1.7	2486	1.2	5752	2.1	1498	1.8
Severity of OIA^†^																		
No sick leave	45,495	59.1	6941	60.2	3593	54.0	2155	47.2	134	52.1	1220	48.9	1337	53.8	2844	49.4	782	52.2
1-3 days	8298	10.8	1336	11.6	683	10.3	571	12.5	40	15.6	377	15.1	381	15.3	863	15.0	189	12.6
4-14 days	11,435	14.8	1672	14.5	1086	16.3	851	18.7	48	18.7	488	19.6	453	18.2	1094	19.0	249	16.6
>14 days	11,795	15.3	1581	13.7	1293	19.4	986	21.6	35	13.6	409	16.4	315	12.7	951	16.5	278	18.6

EU/EFTA: European Union/European Free Trade Association; OIA: Occupational injuries due to accidents

^†^Calculated based on the number of total occurrence of OIA at first appearance

Time since immigration varied depending on the region of birth. The shortest time since immigration (1–5 years) was observed among first-generation immigrants from North America or Oceania (61.9%), Asia (58.5%), and Africa (57.3%), while the longest time since immigration was observed among first-generation immigrants from Nordic or EU/EFTA Member States (34.3%). The highest proportion of reason for immigration due to family reunification or humanitarian reasons was observed amongst first-generation immigrants from the Middle East (85.2%), Africa (81.1%), and European countries outside of the EU/EFTA Member States (64.6%).

Among the first-generation immigrants, a higher proportion of those from non-EU/EFTA European countries were employed in high-risk industries (36.0%). In the current study, the highest proportion of individuals defined as being in full-time employment was native-born workers (49.9%), followed by first-generation immigrants from the Nordic or EU/EFTA Member States (46.6%), while the lowest proportion was found among workers from Africa (22.5%). A larger proportion of second- and first-generation immigrants changed their job in the past year, while the opposite was observed among native-born workers. The proportion of OIA occurrence during the study period was similar across all groups (approximately 1%) and close to half of OIA in all groups did not require any sick leave. This was observed regardless of immigration status.

### Migration status and odds of OIA

Table 2a shows the overall association between origin and OIA among second-generation immigrants and first-generation immigrants. Compared to native-born workers, the odds of OIA was higher among first-generation immigrants (OR 1.41; 95% CI 1.40, 1.42) and second-generation immigrants (OR 1.10; 95% CI 1.09, 1.11) in the unadjusted analysis. After adjusting for sociodemographic factors, time since immigration, and work-related factors the odds of OIA increased among first-generation immigrants (OR 1.59; 95% CI 1.54, 1.63) and second-generation immigrants (OR 1.11; 95% CI 1.10, 1.12), indicating stronger associations.

**Table 2:** Odds ratios (OR) of occupational injuries due to accidents (OIA) for immigrant workers compared to native-born workers based on region of origin and reason for immigration in sweden, 2004-2020

	Crude			Model 1			Model 2			Model 3		
	OR	95% CI		OR	95% CI		OR	95% CI		OR	95% CI	
**a) Overall**												
Native-born (ref.)	1.00			1.00			1.00			1.00		
Second-generation immigrants	1.10	1.09	1.11	1.08	1.07	1.09	1.09	1.08	1.10	1.11	1.10	1.12
First-generation immigrants	1.41	1.40	1.42	1.46	1.46	1.47	1.60	1.55	1.64	1.59	1.54	1.63
**b) Region of birth**												
Native-born (ref.)	1.00			1.00			1.00			1.00		
First-generation immigrants												
Nordic or EU/EFTA Member States	1.11	1.10	1.12	1.18	1.17	1.19	1.30	1.26	1.33	1.28	1.24	1.31
non-EU/EFTA European countries	1.68	1.66	1.70	1.67	1.65	1.69	1.78	1.73	1.83	1.73	1.68	1.79
North America or Oceania	0.73	0.69	0.77	0.92	0.87	0.98	1.03	0.97	1.09	1.12	1.05	1.19
Africa	1.62	1.60	1.65	1.60	1.57	1.62	1.77	1.71	1.83	1.66	1.61	1.71
Asia	1.12	1.10	1.14	1.17	1.15	1.19	1.29	1.25	1.33	1.32	1.28	1.36
Middle East	1.86	1.84	1.88	1.94	1.91	1.96	2.11	2.04	2.17	2.17	2.11	2.24
Other	1.55	1.52	1.57	1.60	1.57	1.63	1.72	1.66	1.78	1.76	1.70	1.81
**c) Reason for immigration**												
Native-born (ref.)	1.00			1.00			1.00			1.00		
First-generation immigrants												
Work	0.62	0.61	0.64	0.79	0.77	0.81				0.86	0.84	0.88
Family reunification or humanitarian	1.69	1.68	1.70	1.70	1.69	1.71				1.74	1.72	1.75
Other	0.79	0.76	0.82	0.96	0.92	1.00				1.06	1.02	1.11

EU/EFTA: European Union/European Free Trade Association; OIA: Occupational injuries due to accidents

CI: Confidence intervals, OR: Odds ratios

Model 1: Adjusted for sex, age and education level

Model 2: Adjusted for sex, age, education level and time since immigration

Model 3: Adjusted for sex, age, education level, time since immigration (only for overall and region of birth), job change in previous year, high-risk industry, and full-time employment

Table 2b shows how the association between first-generation immigrant status and OIA varies by region of birth. In the unadjusted analysis, first-generation immigrants from North America or Oceania had lower odds of OIA compared to native-born workers (OR 0.73; 95% CI 0.69–0.77). Although the odds of OIA were elevated among most immigrant groups, the lowest odds were observed among immigrants from Nordic or EU/EFTA Member States (OR 1.11; 95% CI 1.10–1.12) and Asia (OR 1.12; 95% CI 1.10–1.14). The highest odds were found among immigrants from the Middle East (OR 1.86; 95% CI 1.84–1.88), non-EU/EFTA European countries (OR 1.68; 95% CI 1.66–1.70), Africa (OR 1.62; 95% CI 1.60–1.65), and other regions (OR 1.55; 95% CI 1.52–1.57). These associations were further strengthened in the fully adjusted model.

Table 2c examines the association between reason for immigration and odds of OIA among first-generation immigrants. In the unadjusted analysis, immigrants who arrived for work had lower odds of OIA than native-born workers (OR 0.62; 95% CI 0.61–0.64), while those who immigrated for family reunification or humanitarian reasons had higher odds (OR 1.69; 95% CI 1.68–1.70). Immigrants arriving for other reasons also had lower odds (OR 0.79; 95% CI 0.76–0.82). After adjusting for sociodemographic characteristics, the odds of OIA increased across all groups. In the fully adjusted model, immigrants who arrived for family reunification or humanitarian reasons continued to show the strongest association with OIA (OR 1.74; 95% CI 1.72–1.75). The odds also increased among those who immigrated for work (OR 0.86; 95% CI 0.84–0.88) and for other reasons (OR 1.06; 95% CI 1.02–1.11).

#### Sensitivity analyses

The odds of severe OIA resulting in at least 14 days of sickness absence among first-generation immigrants and second-generation immigrants was similar to the main analyses (Table S2, supplementary materials). The odds of OIA were higher among workers with two immigrant parents than native-born workers and workers with one native-born parent. This remained after adjusting for sociodemographic factors, time since immigration, and work factors (Table S3, supplementary materials). The similar results to the main analyses were found after further adjustment of the fully adjusted models for occurrence of an OIA in the previous year (Table S4, supplementary materials) and in the complete case analysis (Table S5, supplementary materials).

## Discussion

This study investigated the differences in OIA among immigrants relative to native-born workers in Sweden and examined the role of migrant-specific and work factors in these differences. The study found that first-generation immigrants and second-generation immigrants generally have higher odds of OIA compared to native-born workers. The study also found some variations in OIA, particularly among the first-generation immigrants depending on their region of birth and reason for immigration. Migrant-specific factors seem to explain some of the differences while work factors did not. The likelihood of OIA remained at an elevated level among first-generation immigrants. Our findings may be explained by migrant-specific factors, particularly region of birth and reason for immigration. Although controlling for work factors in the analyses did not seem to explain the study findings, the included work factors may give some indication regarding the available type of work and working conditions for jobs performed by immigrants. In line with and adding to the knowledge from previous reports and findings [12, 13], this study further highlights the potential risk factors that contributed to the disparity between immigrant and native-born workers.

Congruent with a recent study [13], this study also found that first-generation immigrants from North America or Oceania initially had a lower odds of OIA than native-born but had higher OIA likelihood compared to native-born after adjusting for time since immigration and work factors. First-generation immigrants from the Middle East, Africa, non-EU/EFTA European countries, and other regions of birth had consistently higher odds of OIA even after adjusting for time since immigration and work factors than native-born across the models. These differences may be explained by dissimilar standard of work safety and culture [14], which may be exacerbated by language barriers, lack of experience, lack of education and/or safety training [8, 11, 16, 31, 32]. However, our data assumes that countries within each region are comparable to each other, possibly ignoring important within-region heterogeneity [14, 16].

The analysis also found that first-generation immigrants who immigrated for family reunification or humanitarian reasons also showed a significantly higher odds of OIA than native-born. First-generation immigrants who immigrated to Sweden for family reunification or humanitarian reasons may also have limited work mobility or have slower labor market integration [33], high chance of mismatch between skills and labor market demands [5], and lack of social network with the native-born population [33]. Altogether, these factors may increase the likelihood of a willingness to perform undesirable work tasks. In turn, immigrants may be more exposed to a more hazardous working condition than native-born workers [32, 34] even when they share the same industry [14, 19]. Some of the reasons may include language barriers, lack of knowledge of OIA practices, limited information about the risks of the job and guidelines on how to report OIA occurrences, and fear of income loss [8, 32]. However, the current study could not account for the work environment that to which the study populations are exposed to, as only information on high-risk occupation based on industry specific rates occupational levels was available.

Meanwhile, first-generation immigrants who immigrated for work had lower odds of OIA than native-born. Presumably, these individuals are employed in high-skilled jobs in low-risk industries, which could be explained by the immigration requirements in Sweden. For instance, a higher threshold minimum salary requirement was implemented for non-Nordic and non-EU/EFTA immigrants [15], which may favor recruitment into a safer and more regulated work environment.

In our sensitivity analysis, where OIA was restricted to only those who were absent for at least 14 days, the study found that second-generation immigrants and first-generation immigrants had higher OIA odds compared to native-born workers. This was similar to the findings in the main analysis, suggesting that the differences in OIA risks between native born and immigrants were unlikely due to differences in reporting. Furthermore, OIA differs among second-generation immigrants. Having one native-born parent appears to be protective, likely due to having better social network with the native population than workers with two immigrant parents [33].

### Strengths and limitations

The use of nationwide high-quality registers in Sweden is considered a major strength of this study. The national coverage of the registers allows us to include most of the working population over time with a low attrition rate. Furthermore, our inclusion criteria enabled an inclusive working population.

This study was also limited by these registers because only individuals with a personal identity number could be included. It was not possible to include unregistered individuals that may be vulnerable to hazardous working conditions. Underreporting also remains a concern in this study, especially for OIA with low severity level. As severe cases of OIA that require sick leave are less likely to be underreported [16, 24], the most severe OIA was retained in the data for cases where an individual has reported multiple injuries during the same year. However, the dichotomization of our outcome variable may have missed important information, such as frequency of less severe OIA when multiple OIAs occur in the same year. Nevertheless, underreporting of OIA has been estimated to be lower in Sweden than other countries and our dataset has previously been substantiated with inpatient and specialized outpatient care data [23]. In the current study, a proxy variable was created to define high-risk industry by comparing diverse industries’ OIA rate to the national average. The broad industry categories used in this study to define those considered as high risk are unlikely to reflect the within industry category variability in occupations that are commonly performed by immigrants, which could explain the low proportion of immigrants in high-risk industries. These categories will also not capture the overrepresentation of immigrant workers in high-risk occupations within industries. Noteworthy is that the odds of OIA increased after adjusting for the limited information available on work factors. This suggests that unobservable factors, such as lived experiences of immigrant workers or migrant specific work factors may be associated with their working conditions [4], resulting in differential risks of OIA. For example, information on the reason for immigration many first-generation immigrants from the Nordic or EU/EFTA Member States was not available due to the free border crossing. Moreover, despite the inclusion criteria, only registered individuals with a personal identity number could be included in the study population. Inevitably, we could not include individuals who are not eligible or have not obtained a personal identity number. Workers who lack a personal identity number (e.g., individuals seeking asylum or pending registration, seasonal workers) may be more willing to work longer hours or perform undesirable work tasks [14, 32]. Still, in the complete case analysis, where first-generation immigrants with missing covariates were excluded, immigrant workers were consistently associated with higher odds of OIA. The data also lacked information on the number of working hours to account for time at risk in the workplace. Therefore, a proxy for full-time employment was created and validated through another statistical source. Nonetheless, it was not possible to rule out potential misclassification.

## Conclusions

Our study found an increased odds of OIA among immigrants, especially among first-generation immigrants, compared to native-born. These associations were observed regardless of how first-generation immigrant groups were defined—by region of birth or reason for immigration, although immigrating due to work reasons may be protective against OIA. Differences in work-related factors only played a small role in these differences. Despite the elevated likelihood of OIA found in this study, our estimates are rather conservative. Register information, particularly among immigrants is limited and poses challenges to estimating the true risks of OIA among immigrants. Our findings emphasize the importance to address the disparities between immigrants and native-born workers in Sweden and to ensure a safer work environment for all. Future research should also consider further examination of differences of the work environment of immigrant workers in Sweden.

## Supplementary Information

Supplementary Material 1

## Data Availability

The data that support the findings of this study are available from Statistics Sweden. Restrictions apply to the availability of these data, which were used under license for the current study and therefore are not publicly available. Data are, however, available from the authors upon reasonable request and with permission from Statistics Sweden.

## Abbreviations

CI: Confidence intervals
EFTA: European Free Trade Association
EU: European Union
ISA: The Information System on Occupational Injuries
LISA: The Longitudinal Integrated Database for Health Insurance and Labor Market Studies
OIA: Occupational injuries due to accidents
OR: Odds ratio
STATIV: The Longitudinal Database for Integration Studies
