# Supplementary material for: Understanding the differences in occupational injuries due to accidents among native-born and immigrant workers in Sweden: a repeated cross-sectional register-based study
Source: Inj Epidemiol. 2025 Sep 8;12:56. doi: 10.1186/s40621-025-00616-7 (PMC12418639; doi:10.1186/s40621-025-00616-7)
Supplement: Supplementary file 1 — Supplementary Material 1 [file 40621_2025_616_MOESM1_ESM.docx]

| **Table S1.** A list of occupational industries with an occupational injury due to accidents (OIA) rate that were 25% higher than the national OIA rate for the years 2005, 2010, 2015, and 2020. |
| --- |
| Mineral extraction |
| Manufacturing |
| Water supply, sewage treatment, waste management |
| Construction |
| Transport and storage |
| Public administration and defence, including compulsory military service |
| Healthcare and social services |
| Note: The annual OIA rate is similar from 2004 to 2020. High-risk industries are only presented with five-year intervals. |

| **Table S2.** Odds ratios (OR) of severe occupational injuries due to accidents (OIA) resulting in sickness absence of at least 14 days for immigrant workers compared to native-born workers based on region of origin and reason for immigration in Sweden, 2004-2020. Results from pooled logistic regression analyses | | | | | | | | | | | | |
| --- | --- | --- | --- | --- | --- | --- | --- | --- | --- | --- | --- | --- |
|  | **Crude** | | | **Model 1** | | | **Model 2** | | | **Model 3** | | |
|  | **OR** | **95% CI** | | **OR** | **95% CI** | | **OR** | **95% CI** | | **OR** | **95% CI** | |
| Native-born (ref.) | 1.00 |  |  | 1.00 |  |  | 1.00 |  |  | 1.00 |  |  |
| Second-generation immigrants | 0.99 | 0.98 | 1.01 | 1.05 | 1.03 | 1.07 | 1.05 | 1.03 | 1.07 | 1.05 | 1.04 | 1.07 |
| First-generation immigrants | 1.66 | 1.64 | 1.68 | 1.73 | 1.71 | 1.75 | 1.77 | 1.66 | 1.89 | 1.66 | 1.56 | 1.78 |
| Model 1: Adjusted for sex, age and education level  Model 2: Adjusted for sex, age, education level and time since immigration  Model 3: Adjusted for sex, age, education level, time since immigration, job change in previous year, high-risk industry, and full-time employment | | | | | | | | | | | | |

| **Table S3.** Odds ratios (OR) of occupational injuries due to accidents (OIA) for immigrant workers compared to native-born workers based on region of origin and reason for immigration in Sweden, 2004-2020, recategorized into workers with one native-born parent and workers with two immigrant parents. Results from pooled logistic regression analyses. | | | | | | | | | | | | |
| --- | --- | --- | --- | --- | --- | --- | --- | --- | --- | --- | --- | --- |
|  | **Crude** | | | **Model 1** | | | **Model 2** | | | **Model 3** | | |
|  | **OR** | **95% CI** | | **OR** | **95% CI** | | **OR** | **95% CI** | | **OR** | **95% CI** | |
| Native-born (ref.) | 1.00 |  |  | 1.00 |  |  | 1.00 |  |  | 1.00 |  |  |
| Second-generation immigrants |  |  |  |  |  |  |  |  |  |  |  |  |
| One native-born parent | 1.05 | 1.04 | 1.06 | 1.04 | 1.03 | 1.05 | 1.05 | 1.04 | 1.06 | 1.06 | 1.05 | 1.07 |
| Two immigrant parents | 1.20 | 1.19 | 1.22 | 1.46 | 1.46 | 1.47 | 1.18 | 1.16 | 1.19 | 1.21 | 1.19 | 1.22 |
| Model 1: Adjusted for sex, age and education level  Model 2: Adjusted for sex, age, education level and time since immigration  Model 3: Adjusted for sex, age, education level, time since immigration, job change in previous year, high-risk industry, and full-time employment | | | | | | | | | | | | |

| **Table S4.** Odds ratios (OR) of occupational injuries due to accidents (OIA) for immigrant workers compared to native-born, 2004-2020 by migration status. | | | |
| --- | --- | --- | --- |
|  | **OR** | **95% CI** | |
| **a) Overall** |  |  |  |
| Native-born (ref.) | 1.00 |  |  |
| Second-generation immigrants | 1.10 | 1.09 | 1.11 |
| First-generation immigrants | 1.54 | 1.50 | 1.58 |
| **b) Region of birth** |  |  |  |
| Native-born (ref.) | 1.00 |  |  |
| First-generation immigrants |  |  |  |
| Nordic or EU/EFTA Member States | 1.25 | 1.22 | 1.29 |
| non-EU/EFTA European countries | 1.67 | 1.62 | 1.72 |
| North America or Oceania | 1.11 | 1.05 | 1.17 |
| Africa | 1.61 | 1.56 | 1.66 |
| Asia | 1.30 | 1.26 | 1.33 |
| Middle East | 2.07 | 2.01 | 2.12 |
| Other | 1.69 | 1.64 | 1.75 |
| **c) Reason for immigration** |  |  |  |
| Native-born (ref.) | 1.00 |  |  |
| First-generation immigrants |  |  |  |
| Work | 0.87 | 0.84 | 0.89 |
| Family reunification or humanitarian | 1.68 | 1.66 | 1.69 |
| Other | 1.07 | 1.02 | 1.11 |
| CI: Confidence intervals; EU/EFTA: European Union/European Free Trade Association; OIA: Occupational injury due to accidents; OR: Odds ratios  Adjusted for sex, age, education level, time since immigration, job change in previous year, high-risk industry, full-time employment, and OIA occurrence in previous year | | | |

| **Table S5.** Odds ratios (OR) of occupational injuries due to accidents (OIA) for immigrant workers compared to native-born workers in Sweden, 2004-2020. Results from pooled logistic regression in complete case analyses. | | | | | | | | | | | | |
| --- | --- | --- | --- | --- | --- | --- | --- | --- | --- | --- | --- | --- |
|  | **Crude** | |  | **Model 1** | |  | **Model 2** | |  | **Model 3** | |  |
|  | **OR** | **95% CI** | | **OR** | **95% CI** | | **OR** | **95% CI** | | **OR** | **95% CI** | |
| Native-born (ref.) | 1.00 |  |  | 1.00 |  |  | 1.00 |  |  | 1.00 |  |  |
| Second-generation immigrants | 1.10 | 1.09 | 1.11 | 1.08 | 1.07 | 1.09 | 1.09 | 1.08 | 1.10 | 1.11 | 1.10 | 1.12 |
| First-generation immigrants | 1.14 | 1.43 | 1.45 | 1.47 | 1.46 | 1.48 | 1.60 | 1.56 | 1.65 | 1.59 | 1.55 | 1.64 |
| CI: Confidence intervals, OR: Odds ratios  Model 1: Adjusted for sex, age and education level  Model 2: Adjusted for sex, age, education level and time since immigration  Model 3: Adjusted for sex, age, education level, time since immigration, job change in previous year, high-risk industry, and full-time employment | | | | | | | | | | | | |
